# Molecular Mechanisms of Inflammation in Sarcopenia: Diagnosis and Therapeutic Update

**DOI:** 10.3390/cells11152359

**Published:** 2022-08-01

**Authors:** Guadalupe Elizabeth Jimenez-Gutierrez, Laura Edith Martínez-Gómez, Carlos Martínez-Armenta, Carlos Pineda, Gabriela Angélica Martínez-Nava, Alberto Lopez-Reyes

**Affiliations:** 1 Laboratorio de Gerociencias, Instituto Nacional de Rehabilitación Luis Guillermo Ibarra Ibarra, Calz México-Xochimilco 289, Tlalpan, Mexico City 14389, Mexico; gejimenez@inr.gob.mx (G.E.J.-G.); laurae.mtzg@gmail.com (L.E.M.-G.); c.armenta1208@gmail.com (C.M.-A.); ameria.justice@gmail.com (G.A.M.-N.); 2 Dirección General, Instituto Nacional de Rehabilitación Luis Guillermo Ibarra Ibarra, Calz México-Xochimilco 289, Tlalpan, Mexico City 14389, Mexico; carpineda@yahoo.com

**Keywords:** sarcopenia, aging, muscle, inflammaging, pyroptosis, imaging

## Abstract

Sarcopenia is generally an age-related condition that directly impacts the quality of life. It is also related to chronic diseases such as metabolic dysfunction associated with diabetes and obesity. This means that everyone will be vulnerable to sarcopenia at some point in their life. Research to find the precise molecular mechanisms implicated in this condition can increase knowledge for the better prevention, diagnosis, and treatment of sarcopenia. Our work gathered the most recent research regarding inflammation in sarcopenia and new therapeutic agents proposed to target its consequences in pyroptosis and cellular senescence. Finally, we compared dual X-ray absorptiometry (DXA), magnetic resonance imaging (MRI), and ultrasound (US) as imaging techniques to diagnose and follow up on sarcopenia, indicating their respective advantages and disadvantages. Our goal is for the scientific evidence presented here to help guide future research to understand the molecular mechanisms involved in sarcopenia, new treatment strategies, and their translation into clinical practice.

## 1. Introduction

Population aging and obesity have become enormous problems for public health, challenging healthcare systems. In 2019, the United Nations Population Prospects and the World Population Ageing indicated that, by the year 2050, the number of people over 60 years will have increased from 1 in 11 individuals to 1 in 6 individuals [1,2]. This situation forces us to have a new social, health, and economic perspective on the elderly [3,4]. One of the many conditions related to aging is sarcopenia, defined by the World European Working Group in Older People 2 (EWGSOP2) as a progressive skeletal muscle disease prone to dramatic outcomes such as falls, fractures, physical disability, and mortality [4,5]. Sarcopenia is characterized by a massive decline in muscle mass and function [6,7] that begins between 30 and 40 years of age, with a 3–8% gradual muscle fiber decline every decade until the 60’s, when the loss compromises the individual’s life [8]. Although sarcopenia is usually related to aging, in *primary* sarcopenia, there are other risk factors, such as cancer, obesity (*sarcopenic obesity*), rheumatic diseases, and malnutrition, that lead to *secondary sarcopenia* [9]. A lack of physical activity or a sedentary lifestyle leads to secondary sarcopenia in earlier life stages [10,11].

The characterization of sarcopenia is essential for clinical care practice. According to the EWGSOP2, not only low muscle mass but also low muscle quality, strength, and physical performance are now accepted as new criteria in the disease diagnosis [5]. Sarcopenia is a multifactorial disease with a wide variety of factors that contribute to its onset, such as lifestyle, physical activity, nutritional habits, reduced satellite cells (myogenic stem cell), changes in muscle protein homeostasis, anabolic resistance, neuromuscular dysfunction, among others.

The EWGSOP2, Asian Working Group for Sarcopenia (AWGS), and other worldwide organizations have established the clinical and research guidelines that help to understand, prevent, detect early, and treat sarcopenia and its dramatic consequences [5,12]. To date there have been no US Food and Drug Administration (FDA)-approved medications for sarcopenia, so therapeutical approaches to overcome sarcopenia could lead to better intervention in healthy aging [13]. Our objective is to provide a molecular perspective on the inflammatory and energetic processes that underlie sarcopenia, analyze non-invasive and low-cost diagnostic tools for early diagnosis, and increase the understanding of treatment.

## 2. Inflammation in Sarcopenia

### 2.1. Inflammaging in Sarcopenia

Aging and lipid metabolism are correlated with the increase in dysfunctional systemic levels of inflammatory molecules (*chronic low-grade inflammation*) known as “*inflammaging*” [14,15], which leads to tissue degeneration and pathogenesis in age-related diseases (type 2 diabetes, osteoarthritis, and sarcopenia) [14,16,17]. Inflammaging is especially connected to sarcopenic obesity, supported by alterations in glucose metabolism, insulin resistance, and oxidative stress, along with exacerbating the production of inflammatory cytokines [18,19,20]. In addition to inflammaging, primary and secondary sarcopenia are also correlated with alterations in lipid metabolism [21]; replacing type II muscle fibers with fat is one of the most prominent characteristics of the disease, contributing to muscle contraction atrophy [22]. The principal inflammatory molecules involved in inflammaging are TNF-α, IL-6, IL-1, and chemokines, which promote the infiltration of inflammatory cells to deteriorate muscle via NF-κB [23]. Lifestyle habits such as physical activity and diet profoundly impact primary and secondary sarcopenia [24,25]; for example, it is well known that a sedentary lifestyle increases the risk of many chronic diseases, including sarcopenia [26]. Moreover, exercise attenuates muscle loss by decreasing the activation of NF-κB [27,28]. Furthermore, diet and nutrition play an important role in the onset of sarcopenia; for example, saturated fat can activate the innate immune system, leading to pro-inflammatory molecule production (IL6 and TNF-α), which, over time, causes insulin resistance [29]. Inflammation triggered by certain foods can be measured by the Dietary Inflammation Index (DII), where higher values of DII represent a greater probability of inflammation. This DII score has been used to establish the inflammatory potential of food; a high DII has been associated with the development of musculoskeletal diseases, including sarcopenia [30]. Therefore, a diet rich in vegetables and fruits is recommended to reduce the risk [31]. In a rat model, a high-fat diet (HFD) caused the accumulation of long-chain fatty acids such as linoleic acid, stearic acid, and vaccenic acid, as well as an increase in the chemokines RANTES, MCP-1, and MIP-2, causing low-grade inflammation and decreased muscle quantity and quality—distinct attributes of sarcopenia [32,33]. The accumulation of palmitic acid derived from a lifestyle with dietary imbalance reduces Akt phosphorylation causing insulin resistance and impairs muscle regeneration [34,35,36]. Moreover, palmitic acid leads to inflammation and cell death by increasing the inflammatory cytokine TNF-α [37], which reveals the lipotoxic effect of high levels of this saturated fatty acid. 

In contrast, omega-3 polyunsaturated fatty acids (such as alpha-linolenic acid (ALA), eicosapentaenoic acid (EPA), and docosahexaenoic acid (DHA)) decrease the adverse effects of sarcopenia by reducing inflammation [38] and improving muscle strength and function in older adults with little effect on muscle mass [39]. In fact, eicosapentaenoic has been shown to have benefits against muscular atrophy caused by palmitic acid accumulation. EPA treatment under lipotoxic stress caused by palmitic acid reduced the rate of muscle protein loss related to the expression of MyoD, myogenin, IGF-II, and IGFBP-5 [37]. These findings support omega-3 polyunsaturated fatty acid administration as a potential therapeutic tool to reduce muscle loss and inflammation linked to accumulated fatty acids in secondary sarcopenia. Likewise, DHA has also recently been found to modulate the ubiquitin–proteasome and the autophagy–lysosome systems, potentially improving muscle integrity and function by decreasing proteolysis and inflammation for sarcopenia [40]. 

Some saturated fatty acids can enhance the signaling pathways involved in the inflammation of macrophages [41]. NF-κB expression, linked to the deregulation of lipid metabolism, is relevant because it activates the NLRP3 inflammasome multiprotein complex that generates a network of immune responses related to local and systemic inflammation [42]. Different signals can lead to the activation of the NLRP3 inflammasome and cause sarcopenia; for example, metabolic dysregulation due not only to obesity but also to insulin resistance [43] leads to hyperglycemia and muscle atrophy via the WWP1/KLF15 pathway [44]. This pathway could be used as a therapeutic target for muscle atrophy and sarcopenia developed by obesity and insulin resistance [45].

### 2.2. Pyroptosis Contributes to Sarcopenia Development

The inflammation-induced activation of the NLRP3 inflammasome can trigger cell death, a process known as pyroptosis [46]. NLRP3 activates the axis caspase-1, which acts upon its substrate gasdermin D (GSDMD), cleaving it [47] and causing multiple membrane pores. These pores allow ion flux and the release of ATP, HMGB1 (high-mobility group box-1), and interleukin (IL)-1β into the cell, provoking membrane rupture and, ultimately, cell death/pyroptosis [48,49,50]. The NLRP3 inflammasome and pyroptosis contribute to muscle dysfunction through a decline in the glycolytic potential and myofiber size [44]. The perspective of the NLRP3 inflammasome reveals the necessity of finding new therapeutical approaches to attenuate inflammation and pyroptosis in sarcopenia. 

**Figure 1:**
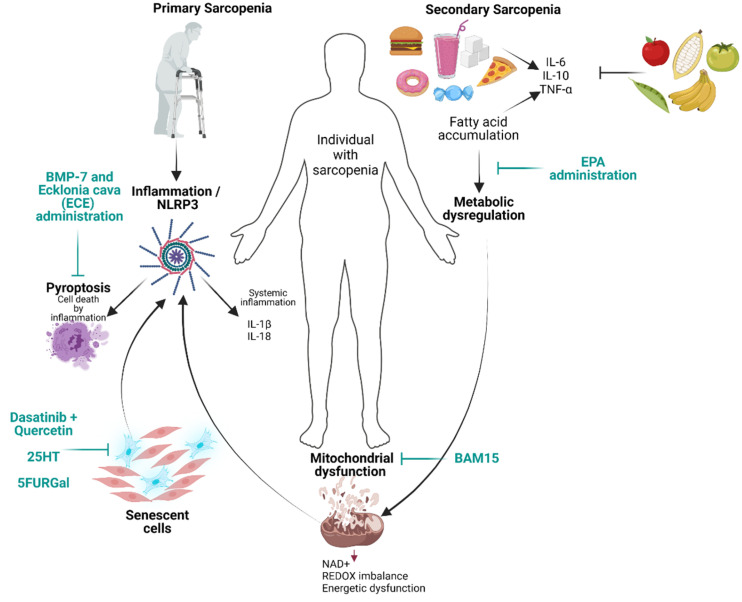
Illustration of factors involved in primary and secondary sarcopenia. New potential agents to treat sarcopenia as well as novel therapeutical approaches which are shown in green color. Created with BioRender.com, accessed on 30 May 2022.

A recent approach is the administration of BMP-7 (bone morphogenetic protein 7), which, in mice, showed the potential to attenuate pyroptosis, inflammation, and muscle atrophy in diabetic muscle myopathy via the inhibition of the HMGB-1 protein. HMGB-1 protein is a potent signaling molecule for inflammation and a key initiator in pyroptosis that binds RAGE or TLR4 and activates other pro-inflammatory factors [51]. Mice with muscle atrophy triggered by the administration of dexamethasone (Dexa) were treated with phlorotannin dieckol (DK) from the brown algae *Ecklonia cava* (ECE) extract. They showed a reduction in protein levels in HMGB-1, NF-κB, and TLR4, key molecules in NLRP3 inflammasome formation (Figure 1) [52]. These therapeutic alternatives offer an alternative to attenuate the effects of inflammation and pyroptosis in sarcopenia.

### 2.3. Inflammaging and Mitochondria

Mitochondrial dysfunction is a common state in sarcopenia related to inflammation [53]. Therefore, mitochondrial quality and homeostasis control are essential to prevent muscle damage. To maintain mitochondrial quality and homeostasis, damaged mitochondria must be removed via mitophagy (organelle-specific autophagy). Conversely, the accumulation of damaged mitochondria and decreased mitophagy are related to sarcopenia [54,55] and are a source of mitochondrial damage-associated molecular patterns mDAMPs (such as mitochondrial DNA (mtDNA), N-formyl peptides, and some lipid species such as cardiolipin). 

Interestingly, inflammation and mDAMPs synergistically contribute to sarcopenia [56]; for example, mDAMPs can activate the Toll-like receptor (TLR) pathway and trigger NF-κB signaling, thus increasing the expression of IL-6 and TNF-α [57]. Damaged mitochondria can activate the NLRP3 inflammasome, triggering the expression of the proteolytic cytokines IL-18 and IL-1β and enhancing inflammation, likely causing pyroptosis (Figure 1) [58]. These reports highlight the importance of the interconnection between mitochondrial dysfunction, triggering potent inflammatory responses that contribute to sarcopenia. In the next section, we analyze mitochondrial morphology and lipid alterations related to sarcopenia development.

### 2.4. Senescence and Its Role in Sarcopenia

Recently, the relevance of cellular senescence in sarcopenia has gained attention due to its relation with inflammaging. Cellular senescence is the permanent cell cycle arrest in response to various stress stimuli or signals that compromise genomic integrity, preventing the proliferation of damaged cells [59]. In addition to cell cycle arrest, cellular senescence is characterized by a highly active metabolic state, telomere shortening, increased ROS, persistent DNA damage, the expression of diverse genes, and the secretion of inflammatory molecules partially regulated by NF-κB, such as IL-1α, IL-6, and IL-8, causing the senescence-associated secretory phenotype (SASP). Over time, this phenotype contributes to the clearance of the same senescent cells by the immune system since accumulation could result in chronic inflammation and changes in the cell microenvironment [59,60,61]. 

The accumulation of senescent cells in muscle correlates with sarcopenia pathophysiology because of the secretion of inflammatory molecules, and the increase in protein degradation results in muscle fiber thinning [62,63,64]. On the one hand, cellular senescence affects the functionality and number of satellite cells (SCs, specific muscle stem cells), which play a fundamental role by switching from their normal quiescent state with a low metabolic rate to an active state to proliferate, differentiate, and form new muscle fibers [65,66]. Subsequent cellular senescence could limit the ability of SC to regenerate and maintain itself by the overexpression of p16 (INK4a), which is the primary inducer of cell cycle arrest in cellular senescence [65]. On the other hand, the SASP significantly contributes to sarcopenia via inflammaging, where the persistent levels of IL-6 harm muscle integrity and function, causing muscle degradation and atrophy [67]. Given the consequences of cellular senescence in muscle degeneration, it is necessary to find new therapeutical strategies to attenuate cellular senescence and its SASP. New approaches have emerged since 2015, when Kirkland’s work group demonstrated the selective depletion of senescent cells in adipose tissue accompanied by decreasing levels of IL-6, IL-8, and CCL2. The precise depletion was achieved by a new type of drugs named senolytics, which include dasatinib and quercetin; the combined treatment can extend the lifespan of aging mice and improve their muscular strength and exercise capacity (Table 1) [68,69]. Preliminary reports suggested that new drugs such as senolytics recover muscular function. In this context, the compound 25HC—an oxygenated derivate of cholesterol that inhibits the CRYAB (Alpha beta-crystallin) protein, which is upregulated in senescent cells—showed significant effects in skeletal muscle. 25HC inhibits the expression of IL-6, a known contributor to muscle atrophy in sarcopenia, attenuating the loss of muscle mass associated with aging in mice and human cultures (Table 1) [70]. Another preliminary report suggested the use of a 5-fluorouridine (5FUR) derivate; 5FURGal kills human senescent cells with multiple benefits such as enhancing cognitive function, lifespan extension, and improvement of muscle tissue and satellite cells (Table 1) [71]. Overall, the connection between SASP and NLRP3 provides a scenario where therapeutic targets against senescent cells (senolytics) and/or the NLRP3 inflammasome could prevent muscle degeneration and sarcopenia, killing two birds with one stone. 

## 3. Mitochondrial Homeostasis in Sarcopenia

Mitochondria play a crucial role in muscle health, function, and homeostasis by acting as cellular energy communicators, regulating intracellular calcium concentrations, and modulating cell proliferation [53]. Therefore, mitochondrial quality and homeostasis control are essential to prevent muscle damage.

### 3.1. Mitochondrial Plasticity and Lipotoxicity in Sarcopenia

Mitochondrial functionality depends on the morphological plasticity regulation between the coordination of two events: (1) *Mitochondrial fusion*, which allows mitochondrial interconnection, signal transmission, and metabolite exchange, is associated with the high utilization of muscle capacity and the regulation of oxidative metabolism and is accompanied by the simultaneous expression of Mfn2 and Opa1 proteins [88]. (2) The opposite event is *mitochondrial fission*, wherein mitochondrial fragmentation is evident, controlling the correct segregation of daughter cells, targeting defective mitochondria for mitophagy elimination and the protein expression of Drp1 and Fis1 [88,89]. Diverse authors have shown a mitochondrial fusion/fission imbalance phenotype in aged mice [55,90]. According to Del Campo, this imbalanced state is observed before sarcopenia is completely developed, contributing to the notion that this preserved phenotype in mitochondrial functionality plays a pivotal role in the pathophysiology of sarcopenia [90]. Earlier this year, the mitochondrial uncoupling agent BAM15 (a protonophore of the respiratory chain) was demonstrated to decrease mitochondrial fission by lowering the expression of Fis1; as a result, increased muscle mass and function, mitochondrial biogenesis quality control, and OXPHOS activity were observed, accompanied by the attenuation of inflammation in sarcopenic mice. These findings provide a new perspective on mitochondrial dynamics in sarcopenia and possible new therapeutical targets [73].

Other mitochondrial disturbances associated with sarcopenia include high sustained levels of triglycerides due to disproportionate diacylglycerol, resulting in a metabolic shift and causing low levels of phosphatidylethanolamine [91,92]. Phosphatidylethanolamine is a key phospholipid for membrane fluidity, inducing a persistent fusion-like phenotype in mitochondrial morphology—this persistent morphological state is a known contributor to sarcopenia development (Figure 1) [91,93]. In sarcopenia, lipid metabolism dysregulation is widespread. Excess triglyceride and HDL are strongly correlated with sarcopenia [94,95]. Furthermore, unbalanced lipid components directly impact mitochondrial morphology and functionality [91]. These mitochondrial changes are major factors that contribute to the age-dependent muscle degeneration observed in sarcopenia [96]. 

### 3.2. Alterations in Energy Metabolism Implicated in Sarcopenia

Understanding the metabolome in sarcopenia could significantly contribute to determining its pathophysiology and treatment. In recent years, diverse metabolomic profiles have been performed, mainly in the skeletal muscle of mice, showing a general perspective on metabolites that could serve as novel targets to diagnose and treat sarcopenia.

Regarding age-related energy dysfunction, a genome-wide transcriptional analysis in individuals of diverse ethnicities with sarcopenia showed mitochondrial energy dysfunction as a major disruption accompanied by an impaired OXPHOS system. As a result, NAD^+^ levels, the principal regulator of REDOX balance, were reduced significantly; likewise, its activity was notably decreased, revealing the essential role of mitochondria in appropriate muscle function independently of ethnicity [97].

On the subject of glycerophospholipids (GPLs) in the gastrocnemius and soleus of FBN-aged rats (Fischer/brown Norway rat model of aging), the metabolic analysis showed a reduction in carnitine [98]. Low levels of carnitine were associated with cardiomyopathy, muscle weakness [99], and mitochondrial dysfunction [100]. Carnitine is an important amino acid essential for energy metabolism due to its role in the mitochondrial 𝛽-oxidation of fatty acids. In recent years, low carnitine levels have been associated with secondary sarcopenia triggered by conditions such as chronic liver disease, liver cirrhosis, and gastrointestinal cancer [101,102,103]. Recently, carnitine levels have been used as a sarcopenic biomarker [103] and a potential candidate to delay muscle deterioration [102,104]; therefore, these metabolomic alterations in FBN-aged rats could be extrapolated to humans with sarcopenia. 

Polyamines are biomolecules composed of multiple amino groups interacting with diverse molecules such as DNA, RNA, and ATP at physiological conditions [105], participating in cell growth and protein synthesis [106]. In early 2006, a reduction in total polyamine levels in the muscle of aged mice was described [107]. Recently, metabolomic analyses in aged mice have shown both a notorious reduction in spermidine and spermine and a lower expression of S-adenosylmethionine decarboxylase [108]. In mice, spermidine, in combination with exercise, reduced the muscular atrophy and sarcopenia via the AMPK-FOXO3a pathway, which resulted in autophagy activation, promoted myogenesis, and reduced D-gal apoptosis [109].

Thus, energy metabolism dysfunction is essential in the pathogenesis of sarcopenia. Knowing the molecular targets participating in the disruption could lead to new therapeutic strategies for sarcopenia.

## 4. Other Therapeutic Approaches for Sarcopenia

### 4.1. Biological Therapy Interventions

Biotherapeutic approaches for sarcopenia could provide a reliable perspective for therapeutic intervention for sarcopenia. One attractive candidate to target sarcopenia is Myostatin (MSTN), a member of the transforming growth factor-beta (TGF-β) superfamily and a potent negative regulator of muscle growth and differentiation [110]. In recent years, diverse strategies in designing monoclonal antibodies against MSTN have shown a significant increase in muscle mass and moderate improvement in muscle function in clinical trials—such as the case of landogrozumab. Other evaluations in individuals with sarcopenia are currently underway [74,75]. For example, another MSTN inhibitor, trevogrumab, enhances muscle mass and function in young and old mice. Meanwhile, others (Stamulumab, Domagrozumab, a novel anti-myostatin peptide PINTA-745, and an anti-myostatin adnectin RG6206) increase muscle mass but fail to improve physical strength in clinical trials [75]. 

Other interesting therapeutical approaches for sarcopenia treatment are testosterone and androgen modulators. Testosterone deficiency is clinically associated with sarcopenia and obesity [76]. Testosterone interacts with the androgen receptor (AR), leading to its nuclear translocation to regulate myogenic expression [77]. In clinical trials, testosterone supplementation showed increased muscle mass and strength in older men. However, studies were terminated due to secondary cardiovascular effects, prostatic hyperplasia, and urinary symptoms [111,112]. Therefore, combined strategies to reduce secondary effects have been proposed as a solution, such as exercise or the combination with finasteride that impedes prostatic hyperplasia [113]. 

As many therapeutical approaches fail to improve muscular strength and function (e.g., some MSTN inhibitors), researchers have seriously considered other drugs that simulate the effects of physical activity on activated protein kinase (AMPK) signaling as attractive targets for sarcopenia, for example, metformin, a common type II diabetes treatment. Metformin activates AMPK [114] and modulates diverse biological processes for muscle, such as glucose uptake, fatty acid oxidation, protein metabolism, autophagy, and mitochondrial function [115]; it also decreases the development of sarcopenia by reducing the inflammatory response by NF-κB [116]. Preliminary trials with metformin combined with exercise have improved resistance training in healthy older adults [78,79].

### 4.2. Natural Compounds

Natural compounds with anti-aging effects have been tested for sarcopenia treatment, such as ursolic acid and pentacyclic triterpene acid fruits, including apple peels and tomatidine (a steroidal alkaloid derived from green tomatoes), which increase the quality of muscle mass and grip strength of mice by reducing the activity of ATF4, a mediator of age-related muscle atrophy [80]. 

Plant flavonoids are important as dietary compounds because of their activities in maintaining good health; in this regard, we want to reference flavanols, the main constituents of cocoa beans. Flavanols such as epicatechin have delayed skeletal muscle degeneration in aged mice by reversing the age-altered expression of extracellular matrix peroxisome proliferator-activated receptors (PPARs), which are master regulators for lipid and glucose homeostasis in muscle [81,82]. Furthermore, epicatechin from cocoa beans improves physical performance consistently with the modulation of biomarkers of sarcopenia by decreasing FoxO1A and MuRF1, regulators of muscle degradation [83]. Epicatechin has also been shown to have anti-inflammatory effects in diverse cell types, such as hepatic and glial cells, but this effect remains to be proved in sarcopenia [117,118].

### 4.3. Vitamins

Vitamin D deficiency and low physical activity strongly correlate with muscle mass, strength, physical performance, and sarcopenia [119]. Some authors have suggested vitamin D as a regulator of mitochondrial health and possible implications in satellite cells activity for muscle regeneration [84]. However, the effects of vitamin D can be indirect in muscle function via its relationship with serum calcium and phosphorus [120,121]. Recent studies have demonstrated that the depletion of vitamin D receptors in mice myocytes directly impacts muscle size and strength, demonstrating the participation of signaling vitamin D in muscle function and size [85]. Another work group showed the increased expression of MuRF1 (Muscle RING-finger protein-1) and FOXO3a in mice with limited physical activity and vitamin D deficiency, implying a synergistic effect of vitamin D, physical activity, and muscle protein degradation in sarcopenia [122]. These studies recommend vitamin D supplementation and physical activity to fight against sarcopenia. 

Vitamin C aids carnitine and collagen biosynthesis [123,124] and has a significant and positive association with muscle. Vitamin C deficiency in mice stimulates the upregulation of ubiquitin ligases, such as atrogin1/muscle atrophy F-box (MAFbx) and MuRF1 [125]. Other recent studies have demonstrated that a higher vitamin C intake is positively correlated with a higher muscle mass in men and women [86,87]. These findings are relevant to future treatments and the prevention of sarcopenia.

## 5. Imaging Based on Inflammation as an Approach for Detection and Follow-Up of Sarcopenia

As mentioned above, diverse factors lead to muscle degeneration manifested as a loss of muscle fibers and mass. The sooner sarcopenia diagnosis is established, the easier it is to prevent more health impairments; that is why diagnosis needs reliable and combinable methods for clinical practice. According to EWGSOP2, diagnosis for sarcopenia should follow a step-by-step procedure starting with a validated measure of muscle strength, commonly by grip strength. If grip strength is lower than the reference, sarcopenia should be considered a probability [126]. The next step in diagnosing sarcopenia is measuring muscle mass and quality [5]. For this approach, there are many tools and techniques, each with its respective flaws or limitations. Currently, there is no single reliable universal tool for clinical practice. In this section, we compare the different tools to diagnose sarcopenia.

Clinical visualization methods represent excellent tools to support the diagnosis and monitoring of various pathological conditions, including sarcopenia [127,128,129]. Biopsy has long offered the visualization of the morphological changes in muscle, the infiltration of adipose cells, and fibrous tissue, among other aspects related to muscle quality; therefore, muscle biopsies have been commonly used to diagnose sarcopenia [130]. However, biopsy is an invasive procedure that can cause discomfort and requires posterior wound care; additionally, a relatively larger sample may be required, and the method is unsuitable for people on anticoagulant treatments (Table 1) [131]. In this regard, imaging tools offer a non-invasive option to evaluate muscle integrity and real-time visualization, which provide the personalized and precise determination of muscle quality. 

The various imaging tools can precisely identify vulnerable people before the development of sarcopenia or the early stages of the condition, thus allowing early treatment. 

Dual X-ray absorptiometry (DXA) has become one of the most common tools to quantify body composition (BC). For sarcopenia diagnosis, this method is based on X-ray transmission across the body at two different spectra, visualizing either bone or soft tissue (e.g., fat mass and lean mass) [132]. This tool estimates muscle mass by linking the appendicular lean soft tissue (ALST) and total-body SM mass in an equation [133]. The use of DXA to measure muscle mass is controversial, while some authors use DXA as a reference tool to diagnose sarcopenia because of its validation versus other more expensive imaging methods such as computer tomography (CT) or magnetic resonance imaging (MRI). Furthermore, the radiation exposure is much lower than in CT [134]. Others point out that using DXA as a reference/gold standard is still premature because of discrepancies related to hydration, thickness of soft tissues, and unclear mathematical equations and algorithms used for the estimation of muscle mass leading to cumulative variations and dangerous expectations in sarcopenia diagnosis [135]. 

Another imaging tool that can help analyze muscle integrity and sarcopenia is magnetic resonance imaging (MRI), which is based on the atomic distribution of the body because of a strong magnetic field [136,137]. The organization of the atoms can vary according to the nature of the molecules; this allows the recognition of diverse tissues based on their magnetic attributes [138]. The MRI is the most advanced tool for identifying sarcopenia and is considered the gold standard for muscle mass quantification, showing muscular quality and fat [5,139]. The MRI variation methods accompanied by nuclear polarization have shown a significant intensification of the MRI signal, allowing the visualization of free radical species in tissues [140]. Dynamic nuclear polarization-magnetic resonance imaging (DNP-MRI) is a variation of the above-described technique used to explore real-time REDOX fluctuation states under local muscle inflammation; this is a magnificent MRI approach to follow up pathologies in real time where inflammation plays a fundamental role (Table 2). However, some disadvantages of MRI and its variations are the high costs and restricted accessibility [141], which is why it is not yet a daily technique used worldwide. 

Since MRI is not always an option in the identification and follow-up of sarcopenia, ultrasound (US) can be used since it provides some advantages. First, US is a much more acceptable tool in terms of costs, accessibility, portability, and patient/analyst ease; second, this imaging tool offers a significant degree of image resolution, allowing a good evaluation of muscular integrity quantification and inflammation [142]. US is based on sound waves that the human ear cannot detect (a frequency between 200 kHz and 1.5 MHz) and provides a signal to visualize the size structure of diverse organs and tissues [143]. The use of US has increased exponentially in recent years to diagnose and follow up on various musculoskeletal conditions, including those related to muscle degeneration associated with inflammation and/or restricted mobility [144]. Recent reports have provided standardization and recommendation for the better operation of US to evaluate diverse muscle mass parameters in more than 30 different muscles, taking advantage of the vast presence of US in clinical practice [145,146,147]. These US muscular parameters are considered “ultrasound biomarkers for sarcopenia” and include muscle thickness, echo intensity, pennation angle, fascicle length, contrast-enhanced assessment of vascularization, and the cross-sectional area, among others. One of the most used parameters is the echo intensity, which expresses the muscle quality in terms of structural changes caused by an increase in intramuscular fat infiltration and connective tissue, which results in a higher echo intensity of the muscle in question; it has also been noted that echo intensity is higher in muscle from older than younger people [148,149]. Likewise, US helps to determine the microstructural characteristics of muscle by measuring the pennation angle and fascicle length, as decreased values of these parameters are observed in sarcopenia [150]. 

One of the main challenges for US to be at the same level as MRI (the gold standard imaging tool) in sarcopenia is the universal standardization of the quantitative and ensembled (and not alone) parameters of muscular changes among diverse muscles to evaluate individuals with sarcopenia. Nonetheless, US might positively impact the daily clinical practice for the early identification and follow-up of this disease (Table 2).

Other non-imaging tools are used to measure the muscle mass in sarcopenia diagnosis, such as bioimpedance analysis (BIA) [151]. This technique is based on an electric current through the body, where the tissues rich in water and electrolytes let electric currents pass more easily than adipose tissue. BIA flaws are influenced by many factors, such as age, hydration, and different devices [152,153]. Thus, this tool should only be used when there is limited access to better options [154].

**Table 2:** Comparison of advantages of imaging tools used for sarcopenia. US, DXA, and MRI have their own perks; nevertheless, US could become a gold standard for diagnosis and follow-up in sarcopenia due to its low cost and almost universal use in clinical practice.

Tool	Advantages	Disadvantages
Biopsy [131,155,156]	Morphological, cellular, and biochemical features in muscle Biobanking practices, tissue manipulation, and individual patient characterization	Invasive technique Contraindicated in high-risk complication patients Patient discomfort Possible poor sample size
DXA [134,135]	Relatively cheap, compared with CT or MRI Rapid technique, noninvasive Allows the visualization of different body compartments (bone or soft tissue) Lower radiation exposure compared to other tools such as CT	Hydration and tissue thickness can alter muscle measurement Even low-radiation exposure needs to be considered No portability Variations in muscle mass due to mathematical equations and algorithms
MRI [157,158,159]	Gold standard for imaging sarcopenia No ionizing irradiation Capable of analyzing images after scanning DNP variation can detect the REDOX state in muscle	High cost Zero portability and not always available in hospitals and clinics Restricted accessibility for some people, such as frail individuals or individuals with metal/electronic devices implanted Image interpretation by a health professional
Ultrasound [128,129,142,145,146,148,160]	Non invasive A set of US parameters can be used as biomarkers for sarcopenia No ionizing irradiation Low cost Portability for easy transportation Adequate for all patients Extensive availability in clinics and hospitals Is possible to interpret images at the moment	Necessity of standardization to establish criteria to diagnose sarcopenia Interpretation of images can be user-dependent Sometimes, restricted use in obese individuals

## 6. Conclusions

From a public health point of view, sarcopenia is a dangerous condition that compromises muscle integrity and directly impacts the quality of life of several groups of individuals, mainly elderly people or those with risky lifestyle behaviors [5,161]. Categorizing sarcopenia into primary or secondary is important to prevent, diagnose, and treat this condition [162]. However, it is not an easy task because the dynamics of sarcopenia depend on many factors that can be intrinsic or extrinsic, such as age, sex, physical activity, dietary intake, comorbidities, etc. [5]. The complex dynamics make it difficult to determine between primary and secondary sarcopenia. Primary sarcopenia is always associated with chronological aging and is exacerbated by diseases or other lifestyle factors and leads to secondary sarcopenia, which is the muscle loss for other causes involved in addition to aging [162].

More information about the molecular basis, onset, and diagnosis of sarcopenia can be used for the better prevention and treatment of the disease. Our work comprises some of the most recent information regarding inflammatory molecular mechanisms underlying sarcopenia; additionally, we reviewed different imaging tools to help diagnose and follow up on the disease. 

Aging and metabolic diseases, such as obesity and type II diabetes, are accompanied by a state of chronic inflammation. One of the diverse mechanisms known to contribute to the onset of sarcopenia is the slight but persistent increase in inflammatory mediators such as IL-1β, IL-6, and TNF-α that simultaneously impact muscle metabolism, causing wasting and loss via the mTOR pathway [163,164,165]. One event that exacerbates the inflammatory state in sarcopenia is senescence via its associated secretory phenotype (SASP). The release of soluble factors such as pro-inflammatory cytokines and chemokines has a paracrine effect on neighbor cells, reinforcing the microenvironment of chronic inflammation and cellular senescence. Another factor involved in the onset of sarcopenia is mitochondrial dysfunction, which brings a series of alterations at different levels; for example, the abnormal mitochondrial morphology caused by an impaired lipid metabolism entails the stiffness of membranes and increases reactive oxygen species in muscle degeneration. Additionally, mitochondria have a close relationship with inflammatory processes that could be exacerbated to cause pyroptosis. Mitochondrial dysfunction in sarcopenia can be expressed as differential patterns depending on the class of muscle with altered levels of intermediates that participate in energetic metabolism, i.e. the significant decrease in REDOX regulators and some cofactors essential for the complete glucose oxidation that enhances muscle deterioration. Therefore, strategies directed to decrease the inflammatory state, the clearance of senescent cells in sarcopenia, or the attenuation of mitochondrial dysfunction (Table 1) are vital to improve the future and quality of life of people with sarcopenia.

## Figures and Tables

**Table 1:** The summary table of new therapeutic agents proposed to treat sarcopenia and muscle atrophy.

Therapeutic Approaches	Target	Benefits
Omega 3/Eicosapentaenoic acid (EPA) [37]	Decreases lipotoxicity caused by palmitic acid accumulation and it is associated with increased levels of molecules implicated in myotube formation	Regenerates skeletal muscle and reduces inflammation
Bone morphogenic protein-7 (BMP-7) [51]	Inhibits the pyroptosis initiator HMGB-1 and lowers the protein expression of inflammasome NLRP3	Amelioration of pyroptosis and sarcopenia
Dasatinib + quercetin [68]	Kill senescent cells and decrease levels of IL-6, IL-8 and CCL2	Improve muscular strength and exercise capacity
25HT [72]	Inhibits the CRYAB protein upregulated in senescent cells	Ameliorates muscle loss
5FURGal [71]	Kills senescent cells	Improves muscle tissue and its satellite cells
Ecklonia cava extract (ECE)/dieckol (DK) [52]	Decrease the NLRP3 formation as well as the expression of HMGB-1	Attenuation of muscle atrophy induced by dexamethasone co- treatment
BAM15 [73]	Mitigates exacerbated mitochondrial fission by lowering the expression of Fis1	Increased muscle mass and mitochondrial quality, accompanied by attenuation of inflammation
Landogrozumab and Trevogrumab [74,75]	Monoclonal antibody that inhibits myostatin	Increased muscle mass, moderate improvement in muscle function
Testosterone [76,77]	Interacts with androgen receptor (AR), leading to its nuclear translocation to regulate myogenic expression	Increase in muscle mass and strength
Metformin [78,79]	Activates AMPK	Modulates glucose uptake, fatty acid oxidation and protein metabolism, autophagy, and mitochondrial function in muscle, decreases the inflammatory response by NF-κB
Ursolic acid and tomatidine [80]	Reduce the activity of ATF4, a mediator of age-related muscle atrophy	Increase quality muscle mass and grip strength in mice
Epicatechin from cocoa [81,82,83]	Regulates age-altered expression of extracellular matrix peroxisome proliferator-activated receptors (PPARs) and decreases FOXO1A and MuRF1	Delayed skeletal muscle degeneration and improved physical performance
Vitamin D [84,85]	Decreases MuRF1 and FOXO3a	Regulator of muscle regeneration
Vitamin C [86,87]	Deficiency stimulates the upregulation of ubiquitin ligases, such as atrogin1/muscle atrophy F-box (MAFbx) and MuRF1	Higher intake of Vitamin C has a positive correlation with higher muscle mass

## Abbreviations

EWGSOP2	World European Working Group in Older People 2
AWGS	Asian Working Group for Sarcopenia
FDA	US Food and Drug Administration
DII	Dietary Inflammation Index
HFD	High-fat diet
ALA	Alpha-linolenic acid
EPA	Eicosapentaenoic acid
DHA	Docosahexaenoic acid
IGF-II	Insulin-like growth factor 2
IGFBP-5	Insulin-like growth factor binding protein-5
NLRP3	NOD-, LRR- and pyrin domain-containing protein 3
WWP1/KLF15	WW domain-containing E3 ubiquitin protein liubiquitin-protein-like factor 15
GSDMD	Gasdermin D
HMGB1	High-mobility group box-1
BMP-7	Bone morphogenic protein-7
DK	Dieckol
ECE	Ecklonia cava
mDAMPs	Mitochondrial damage-associated molecular patterns
mDNA	Mitochondrial DNA
ROS	Reactive oxygen species
SASP	Senescence-associated secretory phenotype
SC	Satellite cells
CRYAB	Alpha beta-crystallin protein
25HC	25-hydroxycholesterol
5FUR	5-fluorouridine
OXPHOS	Oxidative phosphorylation
GPLs	Glycerophospholipids
FBN	Fischer/brown Norway rat
MSTN	Myostatin
AR	Androgen receptor
MuRF1	Muscle RING-finger protein-1
DXA	Dual x-ray absorptiometry
CT	Computer tomography
MRI	Magnetic resonance imaging
US	Ultrasound
BIA	Bioimpedance analysis

## References

[1] Department of Economic and Social Affairs, Population Division (2020). World Population Ageing 2019.

[2] Department of Economic and Social Affairs, Population Division (2019). World Population Prospects 2019: Highlights.

[3] Goates S., Du K., Arensberg M.B., Gaillard T., Guralnik J., Pereira S.L. (2019). Economic Impact of Hospitalizations in US Adults with Sarcopenia. J. Frailty Aging.

[4] Tsekoura M., Kastrinis A., Katsoulaki M., Billis E., Gliatis J. (2017). Sarcopenia and Its Impact on Quality of Life. Adv. Exp. Med. Biol..

[5] Cruz-Jentoft A.J., Bahat G., Bauer J., Boirie Y., Bruyère O., Cederholm T., Cooper C., Landi F., Rolland Y., Sayer A.A. (2019). Sarcopenia: Revised European consensus on definition and diagnosis. Age Ageing.

[6] Rosenberg I.H. (1989). Summary comments. Am. J. Clin. Nutr..

[7] Rosenberg I.H. (1997). Sarcopenia: Origins and clinical relevance. J. Nutr..

[8] Larsson L., Degens H., Li M., Salviati L., Lee Y.I., Thompson W., Kirkland J.L., Sandri M. (2019). Sarcopenia: Aging-Related Loss of Muscle Mass and Function. Physiol. Rev..

[9] Kizilarslanoglu M.C., Kuyumcu M.E., Yesil Y., Halil M. (2016). Sarcopenia in critically ill patients. J. Anesth..

[10] Mijnarends D.M., Koster A., Schols J.M., Meijers J.M., Halfens R.J., Gudnason V., Eiriksdottir G., Siggeirsdottir K., Sigurdsson S., Jónsson P.V. (2016). Physical activity and incidence of sarcopenia: The population-based AGES-Reykjavik Study. Age Ageing.

[11] Du Y., Xu T., Yin Z., Espinoza S., Xie Y., Gentry C., Tian Q., Zhao L.J., Shen H., Luo Z. (2022). Associations of physical activity with sarcopenia and sarcopenic obesity in middle-aged and older adults: The Louisiana osteoporosis study. BMC Public Health.

[12] Chen L.K., Woo J., Assantachai P., Auyeung T.W., Chou M.Y., Iijima K., Jang H.C., Kang L., Kim M., Kim S. (2020). Asian Working Group for Sarcopenia: 2019 Consensus Update on Sarcopenia Diagnosis and Treatment. J. Am. Med. Dir. Assoc..

[13] U.S. Food and Drug Administration (FDA) The Voice of the Patient. https://www.fda.gov/media/108220/download.

[14] Franceschi C., Bonafè M., Valensin S., Olivieri F., De Luca M., Ottaviani E., De Benedictis G. (2000). Inflamm-aging. An evolutionary perspective on immunosenescence. Ann. N. Y. Acad. Sci..

[15] Livshits G., Kalinkovich A. (2019). Inflammaging as a common ground for the development and maintenance of sarcopenia, obesity, cardiomyopathy and dysbiosis. Ageing Res. Rev..

[16] Franceschi C., Campisi J. (2014). Chronic inflammation (inflammaging) and its potential contribution to age-associated diseases. J. Gerontol. A Biol. Sci. Med. Sci..

[17] Zembron-Lacny A., Dziubek W., Wolny-Rokicka E., Dabrowska G., Wozniewski M. (2019). The Relation of Inflammaging With Skeletal Muscle Properties in Elderly Men. Am. J. Mens Health.

[18] Rubio-Ruiz M.E., Guarner-Lans V., Pérez-Torres I., Soto M.E. (2019). Mechanisms Underlying Metabolic Syndrome-Related Sarcopenia and Possible Therapeutic Measures. Int. J. Mol. Sci..

[19] Lim H.S., Park Y.H., Suh K., Yoo M.H., Park H.K., Kim H.J., Lee J.H., Byun D.W. (2018). Association between Sarcopenia, Sarcopenic Obesity, and Chronic Disease in Korean Elderly. J. Bone Metab..

[20] Smeuninx B., McKendry J., Wilson D., Martin U., Breen L. (2017). Age-Related Anabolic Resistance of Myofibrillar Protein Synthesis Is Exacerbated in Obese Inactive Individuals. J. Clin. Endocrinol. Metab..

[21] Collins K.H., Paul H.A., Hart D.A., Reimer R.A., Smith I.C., Rios J.L., Seerattan R.A., Herzog W. (2016). A High-Fat High-Sucrose Diet Rapidly Alters Muscle Integrity, Inflammation and Gut Microbiota in Male Rats. Sci. Rep..

[22] Gueugneau M., Coudy-Gandilhon C., Théron L., Meunier B., Barboiron C., Combaret L., Taillandier D., Polge C., Attaix D., Picard B. (2015). Skeletal muscle lipid content and oxidative activity in relation to muscle fiber type in aging and metabolic syndrome. J. Gerontol. A Biol. Sci. Med. Sci..

[23] Zhang X., Li H., He M., Wang J., Wu Y., Li Y. (2022). Immune system and sarcopenia: Presented relationship and future perspective. Exp. Gerontol..

[24] Abete I., Konieczna J., Zulet M.A., Galmés-Panades A.M., Ibero-Baraibar I., Babio N., Estruch R., Vidal J., Toledo E., Razquin C. (2019). Association of lifestyle factors and inflammation with sarcopenic obesity: Data from the PREDIMED-Plus trial. J. Cachexia Sarcopenia Muscle.

[25] Anton S.D., Hida A., Mankowski R., Layne A., Solberg L.M., Mainous A.G., Buford T. (2018). Nutrition and Exercise in Sarcopenia. Curr. Protein Pept. Sci..

[26] Booth F.W., Roberts C.K., Thyfault J.P., Ruegsegger G.N., Toedebusch R.G. (2017). Role of Inactivity in Chronic Diseases: Evolutionary Insight and Pathophysiological Mechanisms. Physiol. Rev..

[27] Burini R.C., Anderson E., Durstine J.L., Carson J.A. (2020). Inflammation, physical activity, and chronic disease: An evolutionary perspective. Sports Med. Health Sci..

[28] Liu H.W., Chang S.J. (2018). Moderate Exercise Suppresses NF-κB Signaling and Activates the SIRT1-AMPK-PGC1α Axis to Attenuate Muscle Loss in Diabetic db/db Mice. Front. Physiol..

[29] Ralston J.C., Lyons C.L., Kennedy E.B., Kirwan A.M., Roche H.M. (2017). Fatty Acids and NLRP3 Inflammasome-Mediated Inflammation in Metabolic Tissues. Annu. Rev. Nutr..

[30] Cervo M.M., Shivappa N., Hebert J.R., Oddy W.H., Winzenberg T., Balogun S., Wu F., Ebeling P., Aitken D., Jones G. (2020). Longitudinal associations between dietary inflammatory index and musculoskeletal health in community-dwelling older adults. Clin. Nutr..

[31] Bagheri A., Soltani S., Hashemi R., Heshmat R., Motlagh A.D., Esmaillzadeh A. (2020). Inflammatory potential of the diet and risk of sarcopenia and its components. Nutr. J..

[32] Laurentius T., Kob R., Fellner C., Nourbakhsh M., Bertsch T., Sieber C.C., Bollheimer L.C. (2019). Long-Chain Fatty Acids and Inflammatory Markers Coaccumulate in the Skeletal Muscle of Sarcopenic Old Rats. Dis. Markers.

[33] Fellner C., Schick F., Kob R., Hechtl C., Vorbuchner M., Büttner R., Hamer O.W., Sieber C.C., Stroszczynski C., Bollheimer L.C. (2014). Diet-induced and age-related changes in the quadriceps muscle: MRI and MRS in a rat model of sarcopenia. Gerontology.

[34] Yang M., Wei D., Mo C., Zhang J., Wang X., Han X., Wang Z., Xiao H. (2013). Saturated fatty acid palmitate-induced insulin resistance is accompanied with myotube loss and the impaired expression of health benefit myokine genes in C2C12 myotubes. Lipids Health Dis..

[35] Deng Y.T., Chang T.W., Lee M.S., Lin J.K. (2012). Suppression of free fatty acid-induced insulin resistance by phytopolyphenols in C2C12 mouse skeletal muscle cells. J. Agric. Food Chem..

[36] Vlavcheski F., Tsiani E. (2018). Attenuation of Free Fatty Acid-Induced Muscle Insulin Resistance by Rosemary Extract. Nutrients.

[37] Saini A., Sharples A.P., Al-Shanti N., Stewart C.E. (2017). Omega-3 fatty acid EPA improves regenerative capacity of mouse skeletal muscle cells exposed to saturated fat and inflammation. Biogerontology.

[38] Custodero C., Mankowski R.T., Lee S.A., Chen Z., Wu S., Manini T.M., Hincapie Echeverri J., Sabbà C., Beavers D.P., Cauley J.A. (2018). Evidence-based nutritional and pharmacological interventions targeting chronic low-grade inflammation in middle-age and older adults: A systematic review and meta-analysis. Ageing Res. Rev..

[39] Cornish S.M., Cordingley D.M., Shaw K.A., Forbes S.C., Leonhardt T., Bristol A., Candow D.G., Chilibeck P.D. (2022). Effects of Omega-3 Supplementation Alone and Combined with Resistance Exercise on Skeletal Muscle in Older Adults: A Systematic Review and Meta-Analysis. Nutrients.

[40] Lee J.H., Jeon J.H., Lee M.J. (2020). Docosahexaenoic Acid, a Potential Treatment for Sarcopenia, Modulates the Ubiquitin-Proteasome and the Autophagy-Lysosome Systems. Nutrients.

[41] Lee J.Y., Sohn K.H., Rhee S.H., Hwang D. (2001). Saturated fatty acids, but not unsaturated fatty acids, induce the expression of cyclooxygenase-2 mediated through Toll-like receptor 4. J. Biol. Chem..

[42] Boaru S.G., Borkham-Kamphorst E., Van de Leur E., Lehnen E., Liedtke C., Weiskirchen R. (2015). NLRP3 inflammasome expression is driven by NF-κB in cultured hepatocytes. Biochem. Biophys. Res. Commun..

[43] Goossens G.H., Blaak E.E., Theunissen R., Duijvestijn A.M., Clément K., Tervaert J.W., Thewissen M.M. (2012). Expression of NLRP3 inflammasome and T cell population markers in adipose tissue are associated with insulin resistance and impaired glucose metabolism in humans. Mol. Immunol..

[44] McBride M.J., Foley K.P., D’Souza D.M., Li Y.E., Lau T.C., Hawke T.J., Schertzer J.D. (2017). The NLRP3 inflammasome contributes to sarcopenia and lower muscle glycolytic potential in old mice. Am. J. Physiol. Endocrinol. Metab..

[45] Hirata Y., Nomura K., Senga Y., Okada Y., Kobayashi K., Okamoto S., Minokoshi Y., Imamura M., Takeda S., Hosooka T. (2019). Hyperglycemia induces skeletal muscle atrophy via a WWP1/KLF15 axis. JCI Insight.

[46] Huang Y., Xu W., Zhou R. (2021). NLRP3 inflammasome activation and cell death. Cell. Mol. Immunol..

[47] Shi J., Gao W., Shao F. (2017). Pyroptosis: Gasdermin-Mediated Programmed Necrotic Cell Death. Trends Biochem. Sci..

[48] de Vasconcelos N.M., Van Opdenbosch N., Van Gorp H., Parthoens E., Lamkanfi M. (2019). Single-cell analysis of pyroptosis dynamics reveals conserved GSDMD-mediated subcellular events that precede plasma membrane rupture. Cell Death Differ..

[49] Chen X., He W.T., Hu L., Li J., Fang Y., Wang X., Xu X., Wang Z., Huang K., Han J. (2016). Pyroptosis is driven by non-selective gasdermin-D pore and its morphology is different from MLKL channel-mediated necroptosis. Cell Res..

[50] Fink S.L., Cookson B.T. (2006). Caspase-1-dependent pore formation during pyroptosis leads to osmotic lysis of infected host macrophages. Cell Microbiol..

[51] Aluganti Narasimhulu C., Singla D.K. (2021). Amelioration of diabetes-induced inflammation mediated pyroptosis, sarcopenia, and adverse muscle remodelling by bone morphogenetic protein-7. J. Cachexia Sarcopenia Muscle.

[52] Oh S., Yang J., Park C., Son K., Byun K. (2021). Dieckol Attenuated Glucocorticoid-Induced Muscle Atrophy by Decreasing NLRP3 Inflammasome and Pyroptosis. Int. J. Mol. Sci..

[53] Ferri E., Marzetti E., Calvani R., Picca A., Cesari M., Arosio B. (2020). Role of Age-Related Mitochondrial Dysfunction in Sarcopenia. Int. J. Mol. Sci..

[54] Leduc-Gaudet J.P., Hussain S.N.A., Barreiro E., Gouspillou G. (2021). Mitochondrial Dynamics and Mitophagy in Skeletal Muscle Health and Aging. Int. J. Mol. Sci..

[55] Leduc-Gaudet J.P., Picard M., St-Jean Pelletier F., Sgarioto N., Auger M.J., Vallée J., Robitaille R., St-Pierre D.H., Gouspillou G. (2015). Mitochondrial morphology is altered in atrophied skeletal muscle of aged mice. Oncotarget.

[56] Irazoki A., Martinez-Vicente M., Aparicio P., Aris C., Alibakhshi E., Rubio-Valera M., Castellanos J., Lores L., Palacín M., Gumà A. (2022). Coordination of mitochondrial and lysosomal homeostasis mitigates inflammation and muscle atrophy during aging. Aging Cell.

[57] Laforge M., Rodrigues V., Silvestre R., Gautier C., Weil R., Corti O., Estaquier J. (2016). NF-κB pathway controls mitochondrial dynamics. Cell Death Differ..

[58] Zhong F., Liang S., Zhong Z. (2019). Emerging Role of Mitochondrial DNA as a Major Driver of Inflammation and Disease Progression. Trends Immunol..

[59] Hernandez-Segura A., Nehme J., Demaria M. (2018). Hallmarks of Cellular Senescence. Trends Cell Biol.

[60] Ferrucci L., Fabbri E. (2018). Inflammageing: Chronic inflammation in ageing, cardiovascular disease, and frailty. Nat. Rev. Cardiol..

[61] Kirkland J.L. (2013). Inflammation and Cellular Senescence: Potential Contribution to Chronic Diseases and Disabilities with Aging. Public Policy Aging Rep..

[62] da Silva P.F.L., Ogrodnik M., Kucheryavenko O., Glibert J., Miwa S., Cameron K., Ishaq A., Saretzki G., Nagaraja-Grellscheid S., Nelson G. (2019). The bystander effect contributes to the accumulation of senescent cells in vivo. Aging Cell.

[63] Wan M., Gray-Gaillard E.F., Elisseeff J.H. (2021). Cellular senescence in musculoskeletal homeostasis, diseases, and regeneration. Bone Res..

[64] Sugihara H., Teramoto N., Nakamura K., Shiga T., Shirakawa T., Matsuo M., Ogasawara M., Nishino I., Matsuwaki T., Nishihara M. (2020). Cellular senescence-mediated exacerbation of Duchenne muscular dystrophy. Sci. Rep..

[65] Sousa-Victor P., Gutarra S., García-Prat L., Rodriguez-Ubreva J., Ortet L., Ruiz-Bonilla V., Jardí M., Ballestar E., González S., Serrano A.L. (2014). Geriatric muscle stem cells switch reversible quiescence into senescence. Nature.

[66] Montarras D., L’Honoré A., Buckingham M. (2013). Lying low but ready for action: The quiescent muscle satellite cell. FEBS J..

[67] Baar M.P., Perdiguero E., Muñoz-Cánoves P., de Keizer P.L. (2018). Musculoskeletal senescence: A moving target ready to be eliminated. Curr. Opin. Pharm..

[68] Xu M., Pirtskhalava T., Farr J.N., Weigand B.M., Palmer A.K., Weivoda M.M., Inman C.L., Ogrodnik M.B., Hachfeld C.M., Fraser D.G. (2018). Senolytics improve physical function and increase lifespan in old age. Nat. Med..

[69] Zhu Y., Tchkonia T., Pirtskhalava T., Gower A.C., Ding H., Giorgadze N., Palmer A.K., Ikeno Y., Hubbard G.B., Lenburg M. (2015). The Achilles’ heel of senescent cells: From transcriptome to senolytic drugs. Aging Cell.

[70] Limbad C., Doi R., McGirr J., Ciotlos S., Perez K., Clayton Z.S., Daya R., Seals D.R., Campisi J., Melov S. (2022). Senolysis induced by 25-hydroxycholesterol targets CRYAB in multiple cell types. iScience.

[71] Doan L., Paine P., Tran C., Parsons B., Hiller A., Joshua I., Collaco N., Vitari A., Morree A.D., Ishak H. (2020). Targeted Senolytic Prodrug Is Well Tolerated and Results in Amelioration of Frailty, Muscle Regeneration and Cognitive Functions in Geriatric Mice. https://www.researchsquare.com/article/rs-92962/v1.

[72] Limbad C., Doi R., McGirr J., Ciotlos S., Perez K., Daya R., Campisi J., Melov S. (2021). Single-Cell Profiling of Skeletal Muscle Reveals a Novel Senolytic Target: CRYAB. https://assets.researchsquare.com/files/rs-456991/v1_covered.pdf?c=1631865893.

[73] Dantas W.S., Zunica E.R.M., Heintz E.C., Vandanmagsar B., Floyd Z.E., Yu Y., Fujioka H., Hoppel C.L., Belmont K.P., Axelrod C.L. (2022). Mitochondrial uncoupling attenuates sarcopenic obesity by enhancing skeletal muscle mitophagy and quality control. J. Cachexia Sarcopenia Muscle.

[74] Suh J., Lee Y.S. (2020). Myostatin Inhibitors: Panacea or Predicament for Musculoskeletal Disorders?. J. Bone Metab..

[75] Feike Y., Zhijie L., Wei C. (2021). Advances in research on pharmacotherapy of sarcopenia. Aging Med..

[76] Saad F., Röhrig G., von Haehling S., Traish A. (2017). Testosterone Deficiency and Testosterone Treatment in Older Men. Gerontology.

[77] Morley J.E. (2017). Hormones and Sarcopenia. Curr. Pharm. Des..

[78] Long D.E., Peck B.D., Martz J.L., Tuggle S.C., Bush H.M., McGwin G., Kern P.A., Bamman M.M., Peterson C.A. (2017). Metformin to Augment Strength Training Effective Response in Seniors (MASTERS): Study protocol for a randomized controlled trial. Trials.

[79] Walton R.G., Dungan C.M., Long D.E., Tuggle S.C., Kosmac K., Peck B.D., Bush H.M., Villasante Tezanos A.G., McGwin G., Windham S.T. (2019). Metformin blunts muscle hypertrophy in response to progressive resistance exercise training in older adults: A randomized, double-blind, placebo-controlled, multicenter trial: The MASTERS trial. Aging Cell.

[80] Ebert S.M., Dyle M.C., Bullard S.A., Dierdorff J.M., Murry D.J., Fox D.K., Bongers K.S., Lira V.A., Meyerholz D.K., Talley J.J. (2015). Identification and Small Molecule Inhibition of an Activating Transcription Factor 4 (ATF4)-dependent Pathway to Age-related Skeletal Muscle Weakness and Atrophy. J. Biol. Chem..

[81] Phua W.W.T., Wong M.X.Y., Liao Z., Tan N.S. (2018). An aPPARent Functional Consequence in Skeletal Muscle Physiology via Peroxisome Proliferator-Activated Receptors. Int J. Mol. Sci..

[82] Si H., Wang X., Zhang L., Parnell L.D., Admed B., LeRoith T., Ansah T.A., Zhang L., Li J., Ordovás J.M. (2019). Dietary epicatechin improves survival and delays skeletal muscle degeneration in aged mice. FASEB J..

[83] Munguia L., Ramirez-Sanchez I., Meaney E., Villarreal F., Ceballos G., Najera N. (2020). Flavonoids from dark chocolate and (-)-epicatechin ameliorate high-fat diet-induced decreases in mobility and muscle damage in aging mice. Food Biosci..

[84] Latham C.M., Brightwell C.R., Keeble A.R., Munson B.D., Thomas N.T., Zagzoog A.M., Fry C.S., Fry J.L. (2021). Vitamin D Promotes Skeletal Muscle Regeneration and Mitochondrial Health. Front. Physiol..

[85] Girgis C.M., Cha K.M., So B., Tsang M., Chen J., Houweling P.J., Schindeler A., Stokes R., Swarbrick M.M., Evesson F.J. (2019). Mice with myocyte deletion of vitamin D receptor have sarcopenia and impaired muscle function. J. Cachexia Sarcopenia Muscle.

[86] Welch A.A., Jennings A., Kelaiditi E., Skinner J., Steves C.J. (2020). Cross-Sectional Associations Between Dietary Antioxidant Vitamins C, E and Carotenoid Intakes and Sarcopenic Indices in Women Aged 18–79 Years. Calcif Tissue Int.

[87] Lewis L.N., Hayhoe R.P.G., Mulligan A.A., Luben R.N., Khaw K.T., Welch A.A. (2020). Lower Dietary and Circulating Vitamin C in Middle- and Older-Aged Men and Women Are Associated with Lower Estimated Skeletal Muscle Mass. J. Nutr..

[88] Twig G., Elorza A., Molina A.J., Mohamed H., Wikstrom J.D., Walzer G., Stiles L., Haigh S.E., Katz S., Las G. (2008). Fission and selective fusion govern mitochondrial segregation and elimination by autophagy. Embo J..

[89] Iqbal S., Ostojic O., Singh K., Joseph A.M., Hood D.A. (2013). Expression of mitochondrial fission and fusion regulatory proteins in skeletal muscle during chronic use and disuse. Muscle Nerve.

[90] Del Campo A., Contreras-Hernández I., Castro-Sepúlveda M., Campos C.A., Figueroa R., Tevy M.F., Eisner V., Casas M., Jaimovich E. (2018). Muscle function decline and mitochondria changes in middle age precede sarcopenia in mice. Aging (Albany NY).

[91] Pollard A.K., Ortori C.A., Stöger R., Barrett D.A., Chakrabarti L. (2017). Mouse mitochondrial lipid composition is defined by age in brain and muscle. Aging.

[92] Hinkley J.M., Cornnell H.H., Standley R.A., Chen E.Y., Narain N.R., Greenwood B.P., Bussberg V., Tolstikov V.V., Kiebish M.A., Yi F. (2020). Older adults with sarcopenia have distinct skeletal muscle phosphodiester, phosphocreatine, and phospholipid profiles. Aging Cell.

[93] Perreault L., Newsom S.A., Strauss A., Kerege A., Kahn D.E., Harrison K.A., Snell-Bergeon J.K., Nemkov T., D’Alessandro A., Jackman M.R. (2018). Intracellular localization of diacylglycerols and sphingolipids influences insulin sensitivity and mitochondrial function in human skeletal muscle. JCI Insight.

[94] Wang N., Chen M., Fang D. (2020). Relationship between serum triglyceride to high-density lipoprotein cholesterol ratio and sarcopenia occurrence rate in community-dwelling Chinese adults. Lipids Health Dis.

[95] Chung T.H., Kwon Y.J., Shim J.Y., Lee Y.J. (2016). Association between serum triglyceride to high-density lipoprotein cholesterol ratio and sarcopenia in elderly Korean males: The Korean National Health and Nutrition Examination Survey. Clin. Chim Acta.

[96] Alway S.E., Mohamed J.S., Myers M.J. (2017). Mitochondria Initiate and Regulate Sarcopenia. Exerc Sport Sci. Rev..

[97] Migliavacca E., Tay S.K.H., Patel H.P., Sonntag T., Civiletto G., McFarlane C., Forrester T., Barton S.J., Leow M.K., Antoun E. (2019). Mitochondrial oxidative capacity and NAD(+) biosynthesis are reduced in human sarcopenia across ethnicities. Nat. Commun..

[98] Garvey S.M., Dugle J.E., Kennedy A.D., McDunn J.E., Kline W., Guo L., Guttridge D.C., Pereira S.L., Edens N.K. (2014). Metabolomic profiling reveals severe skeletal muscle group-specific perturbations of metabolism in aged FBN rats. Biogerontology.

[99] Wu C., Zhu M., Lu Z., Zhang Y., Li L., Li N., Yin L., Wang H., Song W., Xu H. (2021). L-carnitine ameliorates the muscle wasting of cancer cachexia through the AKT/FOXO3a/MaFbx axis. Nutr. Metab..

[100] Knottnerus S.J.G., Bleeker J.C., Wüst R.C.I., Ferdinandusse S., IJlst L., Wijburg F.A., Wanders R.J.A., Visser G., Houtkooper R.H. (2018). Disorders of mitochondrial long-chain fatty acid oxidation and the carnitine shuttle. Rev. Endocr. Metab. Disord..

[101] Nakano D., Kawaguchi T., Tsutusumi T., Yamamura S., Shigeto K., Hashida R., Koga H., Torimura T. (2020). Alteration of the serum myostatin level following L-carnitine treatment in patients with chronic liver disease: A pilot study. Int. J. Funct. Nutr..

[102] Ohara M., Ogawa K., Suda G., Kimura M., Maehara O., Shimazaki T., Suzuki K., Nakamura A., Umemura M., Izumi T. (2018). L-Carnitine Suppresses Loss of Skeletal Muscle Mass in Patients With Liver Cirrhosis. Hepatol. Commun..

[103] Takagi A., Hawke P., Tokuda S., Toda T., Higashizono K., Nagai E., Watanabe M., Nakatani E., Kanemoto H., Oba N. (2021). Serum carnitine as a biomarker of sarcopenia and nutritional status in preoperative gastrointestinal cancer patients. J. Cachexia Sarcopenia Muscle.

[104] Montesano A., Senesi P., Luzi L., Benedini S., Terruzzi I. (2015). Potential Therapeutic Role of L-Carnitine in Skeletal Muscle Oxidative Stress and Atrophy Conditions. Oxidative Med. Cell. Longev..

[105] Watanabe S., Kusama-Eguchi K., Kobayashi H., Igarashi K. (1991). Estimation of polyamine binding to macromolecules and ATP in bovine lymphocytes and rat liver. J. Biol. Chem..

[106] Miyamoto S., Kashiwagi K., Ito K., Watanabe S., Igarashi K. (1993). Estimation of polyamine distribution and polyamine stimulation of protein synthesis in Escherichia coli. Arch. Biochem. Biophys..

[107] Nishimura K., Shiina R., Kashiwagi K., Igarashi K. (2006). Decrease in polyamines with aging and their ingestion from food and drink. J. Biochem..

[108] Uchitomi R., Hatazawa Y., Senoo N., Yoshioka K., Fujita M., Shimizu T., Miura S., Ono Y., Kamei Y. (2019). Metabolomic Analysis of Skeletal Muscle in Aged Mice. Sci. Rep..

[109] Fan J., Yang X., Li J., Shu Z., Dai J., Liu X., Li B., Jia S., Kou X., Yang Y. (2017). Spermidine coupled with exercise rescues skeletal muscle atrophy from D-gal-induced aging rats through enhanced autophagy and reduced apoptosis via AMPK-FOXO3a signal pathway. Oncotarget.

[110] Argilés J.M., Orpí M., Busquets S., López-Soriano F.J. (2012). Myostatin: More than just a regulator of muscle mass. Drug Discov. Today.

[111] LeBrasseur N.K., Lajevardi N., Miciek R., Mazer N., Storer T.W., Bhasin S. (2009). Effects of testosterone therapy on muscle performance and physical function in older men with mobility limitations (The TOM Trial): Design and methods. Contemp. Clin. Trials.

[112] Dias J.P., Melvin D., Shardell M., Ferrucci L., Chia C.W., Gharib M., Egan J.M., Basaria S. (2016). Effects of Transdermal Testosterone Gel or an Aromatase Inhibitor on Prostate Volume in Older Men. J. Clin. Endocrinol. Metab..

[113] Borst S.E., Yarrow J.F., Conover C.F., Nseyo U., Meuleman J.R., Lipinska J.A., Braith R.W., Beck D.T., Martin J.S., Morrow M. (2014). Musculoskeletal and prostate effects of combined testosterone and finasteride administration in older hypogonadal men: A randomized, controlled trial. Am. J. Physiol. Endocrinol. Metab..

[114] Zhou G., Myers R., Li Y., Chen Y., Shen X., Fenyk-Melody J., Wu M., Ventre J., Doebber T., Fujii N. (2001). Role of AMP-activated protein kinase in mechanism of metformin action. J. Clin. Investig..

[115] Kulkarni A.S., Brutsaert E.F., Anghel V., Zhang K., Bloomgarden N., Pollak M., Mar J.C., Hawkins M., Crandall J.P., Barzilai N. (2018). Metformin regulates metabolic and nonmetabolic pathways in skeletal muscle and subcutaneous adipose tissues of older adults. Aging Cell.

[116] Kanigur Sultuybek G., Soydas T., Yenmis G. (2019). NF-κB as the mediator of metformin’s effect on ageing and ageing-related diseases. Clin. Exp. Pharm. Physiol..

[117] Wu H., Xie Y., Xu Y., Hu Z., Wan X., Huang H., Huang D. (2020). Protective effect of Epicatechin on APAP-induced acute liver injury of mice through anti-inflammation and apoptosis inhibition. Nat. Prod. Res..

[118] Kim J.M., Heo H.J. (2022). The roles of catechins in regulation of systemic inflammation. Food Sci. Biotechnol..

[119] Tieland M., Brouwer-Brolsma E.M., Nienaber-Rousseau C., van Loon L.J., De Groot L.C. (2013). Low vitamin D status is associated with reduced muscle mass and impaired physical performance in frail elderly people. Eur. J. Clin. Nutr.

[120] Girgis C.M., Clifton-Bligh R.J., Hamrick M.W., Holick M.F., Gunton J.E. (2013). The roles of vitamin D in skeletal muscle: Form, function, and metabolism. Endocr. Rev..

[121] Schubert L., DeLuca H.F. (2010). Hypophosphatemia is responsible for skeletal muscle weakness of vitamin D deficiency. Arch. Biochem. Biophys..

[122] Yang A., Lv Q., Chen F., Wang Y., Liu Y., Shi W., Liu Y., Wang D. (2020). The effect of vitamin D on sarcopenia depends on the level of physical activity in older adults. J. Cachexia Sarcopenia Muscle.

[123] Rebouche C.J. (1991). Ascorbic acid and carnitine biosynthesis. Am. J. Clin. Nutr..

[124] Abdullah M., Jamil R.T., Attia F.N. (2022). Vitamin C (Ascorbic Acid). StatPearls.

[125] Takisawa S., Funakoshi T., Yatsu T., Nagata K., Aigaki T., Machida S., Ishigami A. (2019). Vitamin C deficiency causes muscle atrophy and a deterioration in physical performance. Sci. Rep..

[126] Lee S.H., Gong H.S. (2020). Measurement and Interpretation of Handgrip Strength for Research on Sarcopenia and Osteoporosis. J. Bone Metab..

[127] Fairclough M., Prenant C., Ellis B., Boutin H., McMahon A., Brown G., Locatelli P., Jones A.K. (2016). A new technique for the radiolabelling of mixed leukocytes with zirconium-89 for inflammation imaging with positron emission tomography. J. Label. Comp. Radiopharm..

[128] Kaeley G.S., Bakewell C., Deodhar A. (2020). The importance of ultrasound in identifying and differentiating patients with early inflammatory arthritis: A narrative review. Arthritis Res..

[129] Svensson C., Eriksson P., Zachrisson H. (2020). Vascular ultrasound for monitoring of inflammatory activity in Takayasu arteritis. Clin. Physiol. Funct Imaging.

[130] Patel H.P., Cooper C., Sayer A.A. (2012). Percutaneous Muscle Biopsy: History, Methods and Acceptability. Muscle Biopsy.

[131] Wilson D., Breen L., Lord J.M., Sapey E. (2018). The challenges of muscle biopsy in a community based geriatric population. BMC Res. Notes.

[132] Ackermans L., Rabou J., Basrai M., Schweinlin A., Bischoff S.C., Cussenot O., Cancel-Tassin G., Renken R.J., Gómez E., Sánchez-González P. (2022). Screening, diagnosis and monitoring of sarcopenia: When to use which tool?. Clin. Nutr. ESPEN.

[133] Kim J., Wang Z., Heymsfield S.B., Baumgartner R.N., Gallagher D. (2002). Total-body skeletal muscle mass: Estimation by a new dual-energy X-ray absorptiometry method. Am. J. Clin. Nutr..

[134] Scafoglieri A., Deklerck R., Tresignie J., De Mey J., Clarys J.P., Bautmans I. (2013). Assessment of regional adipose tissue depots: A DXA and CT comparison in cadavers of elderly persons. Exp. Gerontol..

[135] Scafoglieri A., Clarys J.P. (2018). Dual energy X-ray absorptiometry: Gold standard for muscle mass?. J. Cachexia Sarcopenia Muscle.

[136] Codari M., Zanardo M., di Sabato M.E., Nocerino E., Messina C., Sconfienza L.M., Sardanelli F. (2020). MRI-Derived Biomarkers Related to Sarcopenia: A Systematic Review. J. Magn. Reson. Imaging.

[137] Chianca V., Albano D., Messina C., Gitto S., Ruffo G., Guarino S., Del Grande F., Sconfienza L.M. (2021). Sarcopenia: Imaging assessment and clinical application. Abdom. Radiol..

[138] Magnetic Resonance Imaging (MRI). https://www.nibib.nih.gov/science-education/science-topics/magnetic-resonance-imaging-mri.

[139] Beaudart C., McCloskey E., Bruyère O., Cesari M., Rolland Y., Rizzoli R., Araujo de Carvalho I., Amuthavalli Thiyagarajan J., Bautmans I., Bertière M.C. (2016). Sarcopenia in daily practice: Assessment and management. BMC Geriatr..

[140] Hyodo F., Ito S., Yasukawa K., Kobayashi R., Utsumi H. (2014). Simultaneous and spectroscopic redox molecular imaging of multiple free radical intermediates using dynamic nuclear polarization-magnetic resonance imaging. Anal. Chem..

[141] Guglielmi G., Bazzocchi A. (2016). Editorial. Eur. J. Radiol..

[142] Özçakar L., Kara M., Chang K.V., Çarl A.B., Akkaya N., Tok F., Chen W.S., Wang T.G., Tekin L., Ulaşl A.M. (2015). Nineteen reasons why physiatrists should do musculoskeletal ultrasound: EURO-MUSCULUS/USPRM recommendations. Am. J. Phys. Med. Rehabil..

[143] Ultrasound. https://www.nibib.nih.gov/science-education/science-topics/ultrasound.

[144] Leeuwenberg K.E. (2019). Muscle Ultrasound in Inflammatory Myopathies: A Critical Review. J. Rheum. Dis. Treat..

[145] Perkisas S., Bastijns S., Baudry S., Bauer J., Beaudart C., Beckwée D., Cruz-Jentoft A., Gasowski J., Hobbelen H., Jager-Wittenaar H. (2021). Application of ultrasound for muscle assessment in sarcopenia: 2020 SARCUS update. Eur. Geriatr. Med..

[146] Perkisas S., Bastijns S., Sanchez-Rodriguez D., Piotrowicz K., De Cock A.M. (2021). Application of ultrasound for muscle assessment in sarcopenia: 2020 SARCUS update: Reply to the letter to the editor: SARCUS working group on behalf of the Sarcopenia Special Interest Group of the European Geriatric Medicine Society. Eur. Geriatr. Med..

[147] Leigheb M., de Sire A., Colangelo M., Zagaria D., Grassi F.A., Rena O., Conte P., Neri P., Carriero A., Sacchetti G.M. (2021). Sarcopenia Diagnosis: Reliability of the Ultrasound Assessment of the Tibialis Anterior Muscle as an Alternative Evaluation Tool. Diagnostics.

[148] Mirón Mombiela R., Vucetic J., Rossi F., Tagliafico A.S. (2020). Ultrasound Biomarkers for Sarcopenia: What Can We Tell So Far?. Semin. Musculoskelet Radiol..

[149] Arts I.M., Pillen S., Overeem S., Schelhaas H.J., Zwarts M.J. (2007). Rise and fall of skeletal muscle size over the entire life span. J. Am. Geriatr. Soc..

[150] Kuyumcu M.E., Halil M., Kara Ö., Çuni B., Çağlayan G., Güven S., Yeşil Y., Arık G., Yavuz B.B., Cankurtaran M. (2016). Ultrasonographic evaluation of the calf muscle mass and architecture in elderly patients with and without sarcopenia. Arch. Gerontol. Geriatr..

[151] Cruz-Jentoft A.J., Sayer A.A. (2019). Sarcopenia. Lancet.

[152] Buckinx F., Landi F., Cesari M., Fielding R.A., Visser M., Engelke K., Maggi S., Dennison E., Al-Daghri N.M., Allepaerts S. (2018). Pitfalls in the measurement of muscle mass: A need for a reference standard. J. Cachexia Sarcopenia Muscle.

[153] Gonzalez M.C., Barbosa-Silva T.G., Heymsfield S.B. (2018). Bioelectrical impedance analysis in the assessment of sarcopenia. Curr Opin. Clin. Nutr. Metab. Care.

[154] Cheng K.Y., Chow S.K., Hung V.W., Wong C.H., Wong R.M., Tsang C.S., Kwok T., Cheung W.H. (2021). Diagnosis of sarcopenia by evaluating skeletal muscle mass by adjusted bioimpedance analysis validated with dual-energy X-ray absorptiometry. J. Cachexia Sarcopenia Muscle.

[155] Invernizzi M., Rizzi M., Carda S., Cisari C., Molinari C., Renò F. (2015). Mini invasive skeletal muscle biopsy technique with a tri-axial end cut needle. Eur. Rev. Med. Pharm. Sci..

[156] Anoveros-Barrera A., Bhullar A.S., Stretch C., Esfandiari N., Dunichand-Hoedl A.R., Martins K.J.B., Bigam D., Khadaroo R.G., McMullen T., Bathe O.F. (2019). Clinical and biological characterization of skeletal muscle tissue biopsies of surgical cancer patients. J. Cachexia Sarcopenia Muscle.

[157] Wang F.Z., Sun H., Zhou J., Sun L.L., Pan S.N. (2021). Reliability and Validity of Abdominal Skeletal Muscle Area Measurement Using Magnetic Resonance Imaging. Acad Radiol..

[158] Peñate Medina T., Kolb J.P., Hüttmann G., Huber R., Peñate Medina O., Ha L., Ulloa P., Larsen N., Ferrari A., Rafecas M. (2021). Imaging Inflammation—From Whole Body Imaging to Cellular Resolution. Front. Immunol..

[159] Eto H., Hyodo F., Kosem N., Kobayashi R., Yasukawa K., Nakao M., Kiniwa M., Utsumi H. (2015). Redox imaging of skeletal muscle using in vivo DNP-MRI and its application to an animal model of local inflammation. Free Radic. Biol. Med..

[160] Stringer H.J., Wilson D. (2018). The Role of Ultrasound as a Diagnostic Tool for Sarcopenia. J. Frailty Aging.

[161] Tzeng P.-L., Lin C.-Y., Lai T.-F., Huang W.-C., Pien E., Hsueh M.-C., Lin K.-P., Park J.-H., Liao Y. (2020). Daily lifestyle behaviors and risks of sarcopenia among older adults. Arch. Public Health.

[162] Lewandowicz A., Sławiński P., Kądalska E., Targowski T. (2020). Some clarifications of terminology may facilitate sarcopenia assessment. Arch. Med. Sci..

[163] Pérez-Baos S., Prieto-Potin I., Román-Blas J.A., Sánchez-Pernaute O., Largo R., Herrero-Beaumont G. (2018). Mediators and Patterns of Muscle Loss in Chronic Systemic Inflammation. Front. Physiol..

[164] Costamagna D., Costelli P., Sampaolesi M., Penna F. (2015). Role of Inflammation in Muscle Homeostasis and Myogenesis. Mediat. Inflamm..

[165] Wang J., Leung K.-S., Chow S.K.-H., Cheung W.-H. (2017). Inflammation and age-associated skeletal muscle deterioration (sarcopaenia). J. Orthop. Transl..

